# Inter-Coder Agreement in One-to-Many Classification: Fuzzy Kappa

**DOI:** 10.1371/journal.pone.0149787

**Published:** 2016-03-02

**Authors:** Andrei P. Kirilenko, Svetlana Stepchenkova

**Affiliations:** The Department of Tourism, Recreation and Sport Management, University of Florida, P.O. Box 118208, Gainesville, FL, 32611–8208, United States of America; University of East Piedmont, ITALY

## Abstract

Content analysis involves classification of textual, visual, or audio data. The inter-coder agreement is estimated by making two or more coders to classify the same data units, with subsequent comparison of their results. The existing methods of agreement estimation, e.g., Cohen’s kappa, require that coders place each unit of content into one and only one category (one-to-one coding) from the pre-established set of categories. However, in certain data domains (e.g., maps, photographs, databases of texts and images), this requirement seems overly restrictive. The restriction could be lifted, provided that there is a measure to calculate the inter-coder agreement in the one-to-many protocol. Building on the existing approaches to one-to-many coding in geography and biomedicine, such measure, fuzzy kappa, which is an extension of Cohen’s kappa, is proposed. It is argued that the measure is especially compatible with data from certain domains, when holistic reasoning of human coders is utilized in order to describe the data and access the meaning of communication.

## 1. Introduction

Reality manifests itself in various types of symbolic representations, including naturally occurring texts like tweets, travel photos, or online reviews. Prior to analysis, such texts need to be subjected to a systematic reduction of the content flow to reformulate them in quantifiable, that is, analyzable terms. One way to create structured data from unstructured texts is to use content analysis, a research method that provides “the objective, systematic, and quantitative description of any symbolic behavior” [1]. However, unless computers are used for coding, content analysis employs literary competent coders who classify original data units according to a given category system; such a procedure, inevitably, has a subjective component to it. Consistency of coding is of crucial importance, as it ensures that inferences made from structured data about the phenomenon under study are valid. Thus, the coding procedure is duplicated by independent coders and their coding outcomes are compared, for which various measures of inter-coder agreement exist; there measures are often referred to as reliability indices. The high level of inter-coder agreement provides assurance in the validity of research results, allows division of coding work among multiple coders, and ensures replicability of the study. The existing methods of agreement estimation, e.g., Cohen’s kappa [2] for the two coders, require that coders map each unit of content into one and only one category from the pre-established set of categories (one-to-one protocol).

However, for certain data domains (e.g., maps, images, or documents in databases), as well as for certain data usage purposes, the requirement to describe a set of data by using one-to-one protocol may be overly restrictive. For example, in the LexisNexis Academic database, one of the world’s largest general news databases, each article is coded with several keywords that indicate topical areas to which the article can be assigned. Thus, a short 290-word article “Burrell Collection is set free to tour abroad” in The Times [3] is tagged in LexisNexis with the following keywords: culture departments (90%), legislative bodies (90%), legislation (90%), museums & galleries (89%), painting (79%), art collecting (79%), international tourism (78%), regional & local governments (76%), city government (76%), building renovation (74%), cities (71%), and approvals (70%). This classification system, which, in fact, represents one-to-many coding, aids in pulling out relevant articles in searches, while a one-to-one system would have left out a number of articles that may be of potential interest to those making the search.

Therefore, this article argues that the restriction to describe data using one-to-one protocol could be lifted, provided that there is a measure to calculate the agreement between the coders in one-to-many classification. To assess the agreement between coders when content is classified into multiple categories, this paper proposes an index, fuzzy kappa, which is an extension of Cohen’s kappa. The approach to calculate fuzzy kappa builds on the methods of fuzzy mathematics and follows the approach firmly established in natural sciences, e.g., in geography and biomedicine.

## 2. Study Background

### 2.1 Measures of agreement: Cohen’s kappa

In the content analysis research, three main unit types are recognized: sampling units, coding units, and context units [4,5]. Sampling unit, e.g., a newspaper article, a photograph, or a tweet, provides a basis for identifying a population of symbolic materials, and its size should adequately represent the phenomenon under study. A coding unit (or content unit) is “the specific segment of content that is characterized by placing it in a given category” ([4], p. 116; identifying the coding unit is one of the most fundamental decisions in a content analysis project [6]). In some cases, coding units can coincide with sampling units but never exceed them. A paragraph, a sentence, or a theme, i.e., a single assertion about some subject matter, as well as words or symbols [7,8], can serve as coding units. Research indicates that coding units differ with respect to consistency of the coding procedure: for example, sentences, paragraphs, and entire documents are usually difficult to classify into a single category [4], which lowers the inter-coder agreement. Lastly, context units encompass the coding units and provide necessary context for making classification decisions. Mapping of coding units into a set of pre-established categories is usually performed by trained human coders and has, inevitably, a subjective component to it.

Establishing reliability of coding is regarded as an essential part of any content analysis [5,9]. Typically, the procedure involves independent processing of the coding units by two human coders with subsequent comparison of the results. The percentage agreement, or raw agreement, that calculates the percentage of identical coding decisions (“hits”) is a simple, however, flawed measure, since in some instances coders may agree purely by chance. Even a simple case of mapping data into only two categories, say, *c*_*1*_ and *c*_*2*_, may carry very high raw agreement when one category (*c*_*1*_) is substantially larger than the other (*c*_*2*_). To illustrate, suppose that two independent coders completely at random classify 10 percent of the data into category *c*_*1*_ and 90 percent into category *c*_*2*_. Then the expectation is that they will jointly classify 0.10 x 0.10 = 0.01 of the sample into *c*_*1*_ and 0.90 x 0.90 = 0.81 into *c*_*2*_. That is, just random classification of the data will obtain raw agreement of 0.01+0.81 = 0.82 or 82 percent [10].

Three indices of inter-rater reliability that account for agreement by chance are arguably the best known: Scott’s pi (π) [11], Cohen’s kappa (κ) [2], and Krippendorff’s alpha(α) [12–14]. To correct for the role of chance agreement, Scott’s pi “uses a joint distribution across two coders. This takes into account not just the number of categories but how these categories are used by the coders”([5], p. 150); the assumption is that, in each category, the distribution of coders’ responses is the same. Cohen’s kappa uses the same conceptual formula but allows coders to have different distributions for the same category. Krippendorff’s alpha, in addition to correcting for the chance agreement, reflects the magnitude of the “misses,” adjusting for the variable’s measurement level. While there is disagreement among scholars on the relative merits of various reliability indicators (e.g., [14–16]), a number of sources report Cohen’s kappa as the most widely used measure of coders’ agreement for categorical data in both social and natural sciences [16–18]. Google Scholar search for “Cohen’s kappa”, “Scott’s pi”, and “Krippendorff’s alpha” returns 48,500, 14,300, and 3,540 results, respectively.

Because of the popularity of Cohen’s kappa and following the developments in geography and biomedicine that employed Cohen’s kappa as the basis for agreement estimation in one-to-many classification (“fuzzy” or “soft” classification), this study based an index that measured inter-coder agreement in one-to-many content analysis protocol on Cohen’s kappa. Further in the article the proposed index is referred to as fuzzy kappa. The authors, however, want to point out that the approach they propose to develop a fuzzy index in one-to-many classification can use other reliability indices as the basis as well, producing fuzzy pi (π), fuzzy alpha (α), etc. The next paragraph provides a brief introduction of Cohen’s kappa.

Cohen's kappa measures the agreement between two coders who each classifies N_u_ items into N_c_ mutually exclusive categories. Cohen’s kappa *κ* is computed as:

$$\kappa =\frac{P^{o}-P^{E}}{1-P^{E}}$$ (1)

where *P*^*o*^ is the observed agreement and *P*^*E*^ is the agreement due to chance, assuming different marginal probabilities of the categories as given by the two coders. For example, suppose two coders A and B are classifying N_u_ coding units into N_c_ categories {$c_{1},\;\ldots \;,c_{N_{c}}$}, and they agreed on $N_{u}^{+}$ coding units. Then the observed (raw) agreement will be equal to the sum of coding units on which they agreed, divided by the number of coding units: $P^{o}=N_{u}^{+}/N_{u}$. Now assume that coder A selected category *c*_1_ A_1_ times, category *c*_2_ A_2_ times, and so on, and coder B selected category *c*_1_ B_1_ times, category *c*_2_ B_2_ times, and so on. The expected (by chance) agreement then will be equal to the sum of products of marginal probabilities for each category: $P^{E}=\sum_{j=1}^{N_{c}}\frac{A_{j}}{N_{u}}\frac{B_{j}}{N_{u}}$. Kappa given by Eq (1) is directly interpretable as “the proportion of joint judgments in which there is agreement, after chance agreement is excluded” [2]. Theoretically, kappa ranges from -1.00 to +1.00; however, as kappa is a measure of agreement, only non-negative values are of practical interest to researchers. The value of zero indicates the agreement at chance level, that is, observed and expected agreement are the same. Greater than chance agreement leads to positive values of kappa. The upper limit of +1.00 indicates perfect agreement between coders. The necessary condition for perfect agreement is that the two coders should have the same marginal probabilities for each category from {$c_{1},\;\ldots \;,c_{N_{c}}$} [2].

### 2.2 Single versus multiple classification

A long-recognized methodological issue in content analysis is “single versus multiple” classification [6,19]. The requirement in one-to-one protocol is that for each coding unit the coder selects a category into which the unit fits best. Yet, in practice, coding unit may have multiple attributes, hence belonging to multiple categories, albeit with a different “strength”. The “strength” of belonging to a certain category may vary across all units in the analysis: “certain words clearly represent a particular category… other words indicate or represent a category less strongly than words in the first group, while still other words seem to belong in more than one category” ([6], p. 36). To describe the data semantically accurately, one of the possible solutions would be weighting the category membership ([19], pp. 207–208); however, this practice is not without limitations, and the prevailing strategy has long been to classify each unit of content in the category where it most clearly belongs.

One-to-one coding is relatively easy to apply when the analyzed content can be broken into units small enough, so that the units fit into no more than a single category, or at least so that a rater is able to intelligently select into which of the multiple categories the unit fits best. However, depending on nature of the data, breaking content into small units of a pre-defined length is no guarantee against generating units that are still difficult to classify in a single category. Using even smaller units, like for example, words, slows down manual coding tremendously. Moreover, the smaller a content unit, the greater the need to employ context units in order to be able to gauge the meaning of the textual segment. Thus, at some point, human coders need to revert from small content units to larger segments, closing the loop and facing the same problem. In addition, for some types of data, e.g., a photograph or a microblogging message such as a tweet, a content unit occurs “naturally” and cannot be “broken” into smaller pieces without altering its holistic meaning. For example, when coding the question from a travel forum “*How much does a hotel cost in Nizhni Novgorod*, *Russia*?” coders would face a hard choice between the two categories, those of “accommodations” and “price.”

A widely practiced solution in such cases is to build a more complex category system. Content analysts define a set of variables (first-tier categories), with each of the variables having several levels (second-tier categories). Thus, a content unit is classified into each first-tier category (with the values of 1/0, corresponding to presence/non-presence of a respective category), within each first-tier category the response is one-to-one, and a reliability indicator is calculated for each variable (first-tier category). Using this approach the researchers can see which variables are the most problematic; however, they end up with a host of reliability indicators varying in magnitude but lack an integrated indicator of how similar coders approximated the data using the given set of categories.

It was the Geographical Information Systems (GIS) applications where the requirement of one-to-one coding scheme was initially found to be overly restrictive and leading to paradoxes. Similar to content analysis, the features of a geographical map result from classifying map’s pixels (coding units) into a given set of geographical features (categories). These features could be forests, wells, or any other geographical object. Starting with Monserud and Leemans [20], Cohen’s kappa is a tool that is frequently used to find agreement between two maps depicting spatially distributed categorical variables. With time, however, it was realized that the interpretation of Cohen’s kappa is severely hindered by over-sensitivity of kappa to small deviations in the maps features. Indeed, following the first law of geography that “everything is related to everything else, but near things are more related than distant things” ([21], p. 236), one would intuitively expect two maps with small differences in the positions of the geographical features to be almost identical. Imagine a checkerboard-colored map with neighboring pixels alternatively colored black or white. A 1-pixel shift of this map would generate two maps with perfect disagreement (κ = -1), even though for practical purposes these maps are indistinguishable. This paradox, however, can be resolved by taking into consideration one’s increasing inability to tell map’s features apart when they are close in location and, thus, to choose a best fitting category while classifying them. That is, since one is unable to draw an exact border between two proximate features on a geographical map, it is only logical to consider a fuzzy transition zone where both features may exist, albeit with a different strength.

Binaghi at al. [22] suggested a method to incorporate the fuzzy sets theory [23] into the error matrix, therefore permitting classification into vague classes, and applied the method to classify a remotely sensed image of the Venice Lagoon. Hagen [24] used similar approach to develop freely available software which computes the agreement between geographical maps accounting for uncertainty in positioning of the maps’ features. It was suggested that before calculating the measure of agreement the maps should be transformed by adding to each of its pixels a fuzzy neighborhood belonging to the same category. The degree of belonging to the same category would decrease with distance. Each pixel of the map could then potentially belong to multiple features (categories), with the strength of belonging represented by the values of its “membership function”. Hagen [24] then used the established mechanism of fuzzy mathematics to compute an extension of Cohen’s kappa, which allowed the elements of a map to share their category with spatially proximate elements. Currently, a suit of GIS computer programs Map Comparison Kit (MCK) is available to compute fuzzy kappa agreement between geographical maps (http://www.riks.nl/mck).

Hagen [24] approach was developed with assumptions formulated for geographically distributed data expressed as “fuzzy location–crisp classification”. When reading a map, one can be uncertain about the exact location of geographical features on the maps (fuzzy location); however he or she is certain that the features do not intersect, i.e. each pixel of the map should be classified to only one category (crisp classification). This formulation limits the approach to applications typical in geography. Dou at al. [25] extended the original “fuzzy location–crisp classification” approach (e.g., [24]) to fuzzy classifications and applied the method to comparisons of MRI images in biomedical research. The approach was further extended to comparison of multiple classifiers by Zuehlke et al. [26], who applied it in automatic recognition of pollen concentrations in the air.

As follows from the aforementioned discussion, the ability to calculate agreement estimates in one-to-many content analysis protocol, analogous to what has been done for problems in other fields, is worth considering. Thus, following pioneering studies on fuzzy Cohen’s kappa [24,25] and building on the methods of fuzzy mathematics, the authors propose a way to calculate fuzzy kappa as a measure of inter-coder agreement in the one-to-many content analysis protocol. In the sections that follow, the authors provide a detailed mathematical description of the proposed indicator and give two examples of studies that used visual images to demonstrate the utility of fuzzy kappa for content analysis research. In the first study, fuzzy kappa is calculated in a case of classical one-to-one coding within a two-tier category system and, thus, can be viewed as integration of multiple crisp indices into one single indicator. The second example illustrates one-to-many coding scheme, when a single unit of analysis, that is, a travel photograph, is coded into several categories; this example considers two scenarios of the “membership function” for categories employed in the analysis. In the Discussion section, the authors discuss applicability of the proposed measure, its implications, and limitations. Finally, in the last section of the paper, software for computing fuzzy kappa that is available for free download is briefly described.

## 3. Fuzzy Kappa

Consider a set of N_u_ coding units U = {u} classified by N_r_ coders. Following the formalization suggested by Dou et al. [25], in the traditional crisp formulation, the coders r_1_, …, r_Nr_ are tasked with classifying each coding unit into a unique category from a set of N_c_ categories C = {c}. For simplification, we assume that two coders participate in the classification (N_r_ = 2); however, the analysis can be extended to any number of coders, with Cohen’s kappa being replaced with Fleiss’ generalization.

The result of the coders’ classification is a collection of tables **A**, one per each coder. In a table $A^{r_{i}}\;\in A$, rows represent coding units, columns represent categories, and the table is filled with zeros and ones, where “1” represents a coding unit classified into a corresponding category. In crisp classification, each row then contains only one “1” element. Consider one coding unit *u*_*i*_, represented by one row in each of the tables. The result of the crisp classification of *u*_*i*_ is a natural number *j* representing the selected category *c*_*j*_ ∈ *C*. This result can be expressed as a function *μ*_*j*_(*u*_*i*_), such as *μ*_*j*_(*u*_*i*_) = 1 if a coding unit *u*_*i*_ belongs to the category *j* and *μ*_*j*_(*u*_*i*_) = 0 otherwise. The requirement to classify each coding unit into one and only one category can then be formalized as ∑_*j*_ *μ*_*j*_(*u*_*i*_) = 1 for all *u*_*i*_ ∈ *U*.

Let us consider a coder who is uncertain into which category to classify a particular coding unit. The traditional crisp analysis requires the coder to make a guess. The result of the fuzzy classification of *u*_*i*_ is a fuzzy number that is not related to any particular category but rather takes all possible values, with the membership function *μ*_*j*_ expressing the coder’s certainty that a particular category *c*_*j*_ was selected. The membership function takes values from 0 to 1, with ∑_*j*_ *μ*_*j*_(*u*_*i*_) = 1 for each particular unit *u*_*i*._ Notice that this constraint is not necessary for the method itself; for example, a coder may suggest that a certain unit fully belongs to multiple categories. E.g., LexisNexis classification of newspaper articles is fuzzy, but does not add to unity. Also notice that we assume that we are using discrete membership function notation since it makes software implementation more transparent; the equations for continuous membership function notation are nearly identical.

Therefore, in the fuzzy classification process, the coders are permitted to classify a unit into multiple categories; the membership function is used to express the coder’s confidence in the selection of a particular category in the following manner:

- $\mu_{j}^{r_{1}}\left(u_{i}\right)=1$ if the coder r_1_ is sure that a coding unit *u*_*i*_ belongs only to the category *j*,
- $\mu_{j}^{r_{1}}\left(u_{i}\right)=0$ if the coder is sure that a coding unit *u*_*i*_ does not belong to the category *j*, and
- 0 < *μ*_*j*_(*u*_*i*_) < 1 if the coding unit u belongs to the category *j* and also to other categories.

**Fig 1:**
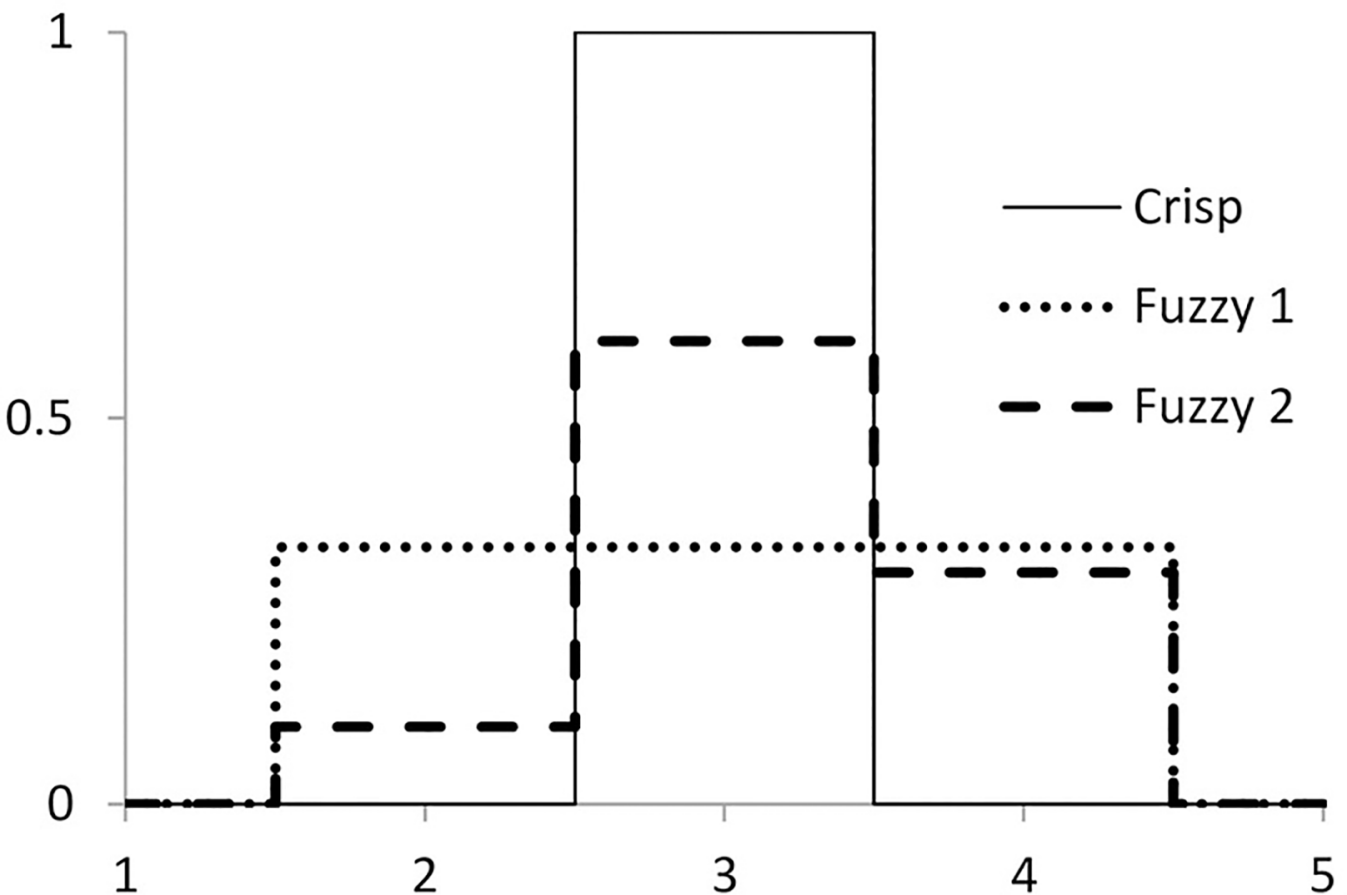
Examples of membership function μ(u): categories 1 through 5. “Crisp” function: coder selects only one category, that of #3. “Fuzzy 1” function: coder selects categories 2, 3, and 4; they are given equal weights. “Fuzzy 2” function: coder selects categories 3 (first choice), 4 (second choice) and 2 (third choice).

The requirement to classify each coding unit into one and only one category is then lifted. Practically, the coders may prefer to interpret the case when a unit is classified into multiple categories as an equal likelihood of each classification or as a decreasing likelihood for each subsequent choice. A selection of either interpretation will then be expressed in a specific [0, 1]-valued membership function (Fig 1).

In the traditional crisp classification, the observed agreement between two coders classifying the same unit *u*_*i*_ is 1 if they classify the unit into same category and 0 otherwise. Using the membership functions introduced above, the observed agreement can be computed as

$$P^{o}=\frac{1}{N_{u}}\sum_{i=1}^{N_{u}}\left(\mu^{r_{1}}\left(u_{i}\right)\cdot \mu^{r_{2}}\left(u_{i}\right)\right)$$ (2)

$$\text{where vector}\;\mu^{r}\left(u_{i}\right)=\left(\mu_{1}^{r}\left(u_{i}\right),\;\ldots \;,\mu_{N_{c}}^{r}\left(u_{i}\right)\right)$$ (3)

and scalar product

$$\left(\mu^{r_{1}}\left(u_{i}\right)\cdot \mu^{r_{2}}\left(u_{i}\right)\right)=\sum_{j=1}^{N_{c}}\left(\mu_{j}^{r_{1}}\left(u_{i}\right)\mu_{j}^{r_{2}}\left(u_{i}\right)\right)=\{\begin{array}{l} 1,\;\text{if}\;\mu^{r_{1}}\left(u_{i}\right)\equiv \mu^{r_{2}}\left(u_{i}\right)\\ \;0,\;\text{otherwise} \end{array}.$$ (4)

Note that $\left(\mu^{r_{1}}(u_{i})\cdot \mu^{r_{2}}(u_{i})\right)$ simply designates an intersection of category selection by two raters, so that the observed agreement is a sum of same choices by the raters (i.e. the choices which intersection = 1) divided by the total number of units. For the fuzzy approach, the observed agreement is no longer defined by the identity of the membership functions for both coders: even when the raters do not completely agree on their classification, their partial agreement should also be accounted for. That is, the category selections may partially overlap. Correspondingly, Eqs (2)–(4) for observed agreement are modified as:

$$P^{o}=\frac{1}{N_{u}}\sum_{i=1}^{N_{u}}\sum_{j=1}^{N_{c}}\left(\mu_{j}^{r_{1}}(u_{i})\;\Lambda \;\mu_{j}^{r_{2}}(u_{i})\right)$$ (5)

(see [25]). Here, binary operation Λ is a t-norm, which is a generalization of intersection. There is a family of functions that confirm the t-norm definition; Zuehlke et al. [26] tested three of the most used functions (*min*, *product*, and *Luka*) with his fuzzy kappa application in brain imagery and recommended using the *min* t-norm:

$$\mu_{j}^{r_{1}}\left(u_{i}\right)\;\Lambda \;\mu_{j}^{r_{2}}\left(u_{i}\right)=min\left(\mu_{j}^{r_{1}}(u_{i}),\mu_{j}^{r_{2}}(u_{i})\right).$$ (6)

The expected agreements for fuzzy and ordinary crisp classifications are computed similarly. For the crisp approach, under the assumption of independence, the probability that a particular unit of coding is classified into the same unique category *j* by both coders is the product of the probability $p_{j}^{E,r_{1}}$ that coder r_1_ selected category *j* and the probability $p_{j}^{E,r_{1}}$ that coder r_2_ selected category *j*, so that the expected agreement is a

$$P^{E}=\sum_{j=1}^{N_{c}}\sum_{\mu_{j}^{r_{1}}=0}^{1}\sum_{\mu_{j}^{r_{2}}=0}^{1}p_{j}^{E,r_{1}}p_{j}^{E,r_{2}}\left(\mu_{j}^{r_{1}}\mu_{j}^{r_{2}}\right)\;=\;\sum_{j=1}^{N_{c}}p_{j}^{E,r_{1}}p_{j}^{E,r_{2}}\;,$$ (7)

with the double sum computed over all values, i.e. 0 and 1, of the membership function.

The probability that the coder r_i_ selected category *j* is computed as the total number of times the coder r_i_ selected category *j* divided by N_u_:

$$p_{j}^{E}=\frac{1}{N_{u}}\sum_{i}\mu_{j}\left(u_{i}\right).$$ (8)

Following [25], for fuzzy approach, Eq (8) stays and Eq (7) transforms into

$$P^{E}\;=\;\sum_{j=1}^{N_{c}}\sum_{\mu_{j}^{r_{1}}=0}^{1}\sum_{\mu_{j}^{r_{2}}=0}^{1}p_{j}^{E,r_{1}}p_{j}^{E,r_{2}}(\mu_{j}^{r_{1}}\;\Lambda \;\mu_{j}^{r_{2}}),$$ (9)

where t-norm operator Λ can be defined by (6). Notice that (9) takes into account partial intersection of the categories selected by the raters; if classification is crisp, then $\mu_{j}^{r_{1}}\;\Lambda \;\mu_{j}^{r_{2}}=\mu_{j}^{r_{1}}\mu_{j}^{r_{2}}$ and Eq (9) is identical to (7).

Note that the Eqs (5) and (9) for observed and expected agreement in “soft” classification become, respectively, Eqs (2) and (7) in the “crisp” context, that is, both crisp and fuzzy measures return identical values when used with one-to-one classification or when the weight of the subsequent coder choices is set to zero in the fuzzy context. Hence, in this case, both the crisp and fuzzy formulations are identical when fitted into the expression of Cohen’s kappa (1).

## 4. Applicational Examples

Application of fuzzy kappa is demonstrated using two examples of content analysis of visuals involving two coders. Table 1 shows values of crisp and fuzzy kappa indicators calculated in each application. Confidence intervals for crisp and fuzzy indices were calculated using the bootstrap method [5,13].

**Table 1:** Inter-coder agreement indices: Examples 1 and 2. For data see support information files S1 and S2 Zip Archives.

Examples		Crisp kappa				Fuzzy kappa			
Number of categories		P^0^	P^E^	*κ*	CI*	P^0^	P^E^	*κ*	CI*
1. Russia	30	-	-	-	-	0.90	0.53	0.79	0.76–0.81
People	5	0.95	0.43	0.90	0.86–0.95	*0*.*95*	*0*.*43*	*0*.*90*	*0*.*86–0*.*95*
Nature Landscape	2	0.99	0.80	0.95	0.89–1.01	*0*.*99*	*0*.*80*	*0*.*95*	*0*.*89–1*.*01*
Place	3	0.85	0.39	0.75	0.68–0.82	*0*.*85*	*0*.*39*	*0*.*75*	*0*.*68–0*.*82*
Space	4	0.73	0.28	0.62	0.55–0.69	*0*.*73*	*0*.*28*	*0*.*62*	*0*.*55–0*.*69*
Transport & Infr	2	0.96	0.68	0.86	0.78–0.94	*0*.*96*	*0*.*68*	*0*.*86*	*0*.*78–0*.*94*
Activities	5	0.85	0.50	0.70	0.62–0.78	*0*.*85*	*0*.*50*	*0*.*70*	*0*.*62–0*.*78*
Season	3	0.96	0.57	0.91	0.85–0.96	*0*.*96*	*0*.*57*	*0*.*91*	*0*.*85–0*.*96*
Architecture	2	0.92	0.53	0.84	0.77–0.90	*0*.*92*	*0*.*53*	*0*.*84*	*0*.*77–0*.*90*
Heritage	4	0.91	0.60	0.76	0.68–0.85	*0*.*91*	*0*.*60*	*0*.*76*	*0*.*68–0*.*85*
2. Peru	20	-	-	-	-	0.84	0.14	0.81	0.75–0.87
First selection only	20	0.79	0.12	0.76	0.67–0.85	*0*.*79*	*0*.*12*	*0*.*76*	*0*.*67–0*.*85*

* 95% confidence interval.

NB: Fuzzy kappa for single categories (in *italic*) equals crisp kappa.

### 4.1 Example 1: Tourists’ Photographs of Russia

The study [27] examined whether destination photographs made by tourists and posted online are reflective of culture to which these tourists belong. The study conducted a comparative content analysis of American and Korean photographs of Russia collected from Flickr (American set, 637 photos) and Korean travel blogs (584 photos). Each photo was regarded as a single unit of content and coded along the following dimensions: PEOPLE (single, group, few random, or crowd), NATURE LANDSCAPE, PLACE (urban area or rural area), SPACE (tourist, residential, or private), TRANSPORT & INFRASTRUCTURE, ACTIVITIES (leisure, outdoors, way of life, or on the streets), SEASON (cold/snow or greenery), ARCHITECTURE, and HERITAGE (arts & culture, history, or state power). Thus, each dimension was, in fact, a first-tier category with several levels (second-tier categories). The total number of the second-tier categories, including “not present,” was 30. For each photograph, coders went through categories in the order they were laid out in Excel coding sheet (Table 2), and every image was classified on each of the nine first-tier categories by choosing the best fitting value (the second-tier category).

**Table 2:** One-to-one coding sheet.

ID	PEOPLE	NATURE LAND	PLACE	SPACE	TRANS/ INF	ACTIV	SEASON	ARCHIT	HERIT
1001	2	0	1	2	0	1	2	0	0
1002	0	0	2	2	0	0	2	1	0
1003	0	0	0	0	0	0	0	1	0
1004	3	0	1	1	0	1	2	0	2
1005	0	0	0	0	0	0	0	1	0
1006	0	0	1	2	0	0	0	0	0
1007	2	0	1	1	0	1	2	1	0

The reliability analysis was conducted by two coders for a subsample of 274 photographs. Since classification within each first-tier category is one-to-one, nine crisp kappa indices were obtained, along with their confidence intervals. Crisp indices ranged from 0.62 (SPACE) to 0.95 (NATURE LANDSCAPE) (Table 1). In calculating fuzzy kappa, 30 second-tier categories become the primary categories. A photo picturing people in Moscow in a rush hour on a spring day can be coded as Urban area, Residential, Crowds, On the streets, Greenery, that is, the coding protocol becomes one-to-many, and fuzzy kappa can be used. Fuzzy kappa was calculated as an integral indicator of inter-coder reliability across 30 second-tier categories and was equal to 0.79.

### Example 2: Tourists’ Photographs of Peru

The study [28] compared destination images of Peru as posted by the country’s Destination Marketing Organization on the official tourism website (530 images) and by tourists to Peru on Flickr (500 images). Each photo was regarded as a single unit of content. Twenty categories to describe the set of images were developed; ten most frequent ones were Nature & Landscape, People, Archaeological Sites, Way of Life, Traditional Clothing, Outdoor/Adventure, Architecture, Wild Life, Art Object, and Tourist Facilities. The nature of the data was such that one, two, or three categories were the most salient in most cases. For example, a colony of birds in the Parakas Reserve exemplified Wild Life; the remnants of Inca structures set in a magnificent mountain landscape–Archeological Site and Nature & Landscape categories; and a woman in a distinctly colored dress mending clothes on the reed island of Puno–People, Way of Life, and Traditional Clothing. Therefore, it was decided to describe the data with no more than four categories for each photograph: one to three “core” categories and one or two “residual” categories, if necessary. Example of a coding sheet is presented in Table 3.

**Table 3:** One-to-many coding sheet.

ID	Cat1	Cat2	Cat3	Cat4
63	WL	FL		
70	PP	TC	FR	
71	NL	PP	OA	
72	A			
114	NL	PP	OA	TF
135	DA	NL		

A formal reliability study was conducted with 100 randomly selected images from each set of photographs, DMO and Flickr. As the results were similar for both sets, reliability indicators are provided for the DMO set only to save space. It was observed that in the coding process, the raters did tend to decide on the most salient categories first and then to move to less prominent “residual” categories. However, in situations of equally salient categories (e.g., an archaeological site in the nature setting), the order of the categories reflected the coder’s preference. The raw percentage agreement in each category (present/not present) was higher than 0.90 for all categories. Fuzzy non-weighted kappa was 0.81 (Table 1). Weighted kappa, with weights assigned arbitrary as 4, 3, 2, and 1, had the value of 0.80. The crisp kappa was calculated for the hypothetical “what if” scenario: if the coders were forced to choose only one category, what would the crisp kappa have been? It was calculated as 0.76.

## 5. Discussion

Collected unstructured data represent the objective reality that researchers want to study and understand. Unless computer-assisted content analysis is used for coding, content analysts must rely upon pooled judgments of human coders to transition from the unstructured to structured data format. The category system employed for coding should facilitate structuring the data in such a way that it still represent the phenomenon under study and can aid researchers in answering their question of interest. The category system should also be conducive to consistent application by independent coders. As the nature of the data is usually beyond the investigator’s control, opportunities to enhance inter-code agreement are generally restricted to disambiguation of the set of categories and coders training. It is often the case in content analysis that the coders “calibrate” their assessments prior to reliability studies and “reconcile” found disagreements after the reliability indicators are calculated. While relentless training and ceaseless explication of coding rules increase consistency of data coding, such procedures in our view do not facilitate replicability of research and, as a result, the validity of research conclusions. Within the one-to-many coding approach, the training time is reasonable and the use of categories is more or less self-evident. Furthermore, while for some types of data more consistent one-to-one coding may be achieved by manipulating the size of the coding units, by splitting content into tinier and tinier bits, analysts take upon themselves the role of a computer, which defeats the purpose of using human coders: the main argument of using human coders is the interpretive power of human intelligence, which can produce a higher level of content understanding.

The capacity of human coders to conduct holistic data evaluations underlines the argument for using fuzzy kappa as an integrative indicator in one-to-many protocol. Studies in cognitive psychology recognize two different approaches of mental evaluations–holistic and analytical [29,30]. The holistic approach attends to the object in its entirety and in connection to other objects, it makes relatively little use of categories and formal logic and relies on “dialectical” reasoning. Example 2 is a holistic one-to-many procedure: the coder looks at the photo and describes it in terms of its most salient categories, adding one or two residual categories, if necessary. It is known that the human brain can do holistic evaluations of high degree of complexity almost instantaneously (e.g., [31]). In our own experience, the holistic format is very conducive to falling in the state of flow [32], or optimal experience, which is characterized by high concentration, speed, seamless transition between steps of the process and, as a result, high degree of consistency of the analysis. Unlike the holistic approach, analytical evaluations focus on the object’s attributes and the categories to which the object belongs and uses rules and formal logic to understand the object’s behavior. In the provided applications, Example 1 is a two-tier one-to-one analytical protocol, when the coder goes through the set of categories one by one, most likely in a predetermined order, and records whether the coding unit possesses the attribute symbolized by the respective category and at what level. In this case, fuzzy kappa serves as a summative inter-coder agreement measure, stepping into place of nine crisp indices (Table 1).

Comparisons of crisp and fuzzy indices provide additional information about nature of the data and the coding process. Higher values of fuzzy kappa indicate that coders encounter coding units that they tend to classify into the same combinations of categories. In the Peru study (Example 2), such categories frequently were Nature & Landscape and Archaeological Sites; Nature & Landscape, People, and Outdoor Activities; or People, Traditional Clothing, and Festivals & Rituals. In this study, the one-to-many protocol produced a higher agreement between the coders (0.81) than a hypothetical one-to-one protocol (0.76). The authors would speculate that the set of images under the study can be described more consistently when coders are allowed to choose several categories rather than just one. Weighted kappa of 0.80, while only marginally lower than non-weighted fuzzy kappa, may indicate that ambiguity exists in arranging the residual categories in the order of their importance. Yet, various scenarios need to be examined in order to better understand behavior of fuzzy kappa, its statistical properties, and what it can tell us about the coding process. One particular area of research would be to establish a “qualitative” scale for interpreting the inter-coder agreement in one-to-many protocol (that is, low, moderate, high, or very high) based on the magnitude of fuzzy kappa values.

We would like to address three particular concerns with respect to one-to-many classification and the proposed fuzzy inter-coder agreement indicator. First, if classification of coding units into multiple categories is allowed, why not to code each unit in all of them? Interestingly, nothing precludes analysts from doing just that, provided that they also decide on the strength of the membership function for each category in every unit they code. In practice, though, just a few categories are usually enough to accurately describe each unit of content, with weights assigned as equal, or in the progressively diminishing order, or in some other way, as the researcher thinks fit. One-to-many coding, however, may lead to categories that lack statistical independence: the frequencies of categories used do not add to the number of units coded and the frequencies of individual categories are no longer comparable. Traditional two-tier content analysis avoids this problem by not including the first- and second-tier categories in the same analysis (e.g. factor analysis); thus keeping the first- and second-tier categories statistically independent ([19], pp. 207–208). In the Peru study (Example 2) statistical independence of categories is under question, since certain categories have a tendency to occur together. While individual categories are no longer compatible within one set of data, two sets of data of the same nature can still be compared: for example, in the Peru study, it was found that Flickr sample had more images of how Peruvian people live than the DMO sample (category Way of Life). Additionally, the strength of association of any two particular categories (co-occurrences of categories) can be analyzed against the “null hypothesis” assumption of categories’ independence (see procedure in [28]). Another practical advantage of one-to-many classification in Example 2 would be the ease of pulling up from the data all relevant images reflecting a particular category and arranging those image in order of strength of that category’s membership function.

Finally, there is an aspect of the utility of fuzzy kappa, since traditional crisp indicators can be used if one-to-many classification is reformulated in terms of one-to-one classification. For example, in coding a set of photographs, the question “to which of the following four categories does this photograph belong?” can be reformulated as “mark all categories to which the photograph belongs with 1, and the rest of the categories with 0”. Then coding of a photograph returns a binary string with 2^4^ = 16 possible values. Accepting these values as new categories, the original one-to-many classification to four categories can be replaced with a one-to-one classification to sixteen categories. There are several limitations in this approach. First, if two coders partially agree on coding a photograph, the one-to-one approach nevertheless will count this photograph towards the number of “misses”. Application of specific distance measures from vector mathematics may reduce this problem; however this would require further modification of the reliability indices. Further, the vector approach precludes the raters from expressing the degree a coding unit belongs to different categories through ranking their preferences, for example. Finally, the number of re-formulated categories grows exponentially: just four original categories produce sixteen binary combinations; mapping the photographs into ten categories would require 1024 categories in one-to-one classification. Fuzzy kappa is free from these limitations.

The difference between fuzzy kappa and Cohen’s weighted kappa [33] should be noted. Cohen’s weighted kappa was designed as a provision of weighing the amount of disagreement between the choices. For example, for two raters classifying landscape photographs, the amount of disagreement between rater A mapping a photograph into category “deciduous forest” and rater B mapping the same photograph into category “coniferous forest” would be smaller in comparison to rater B mapping the photograph into category “agricultural land”. Notice that the raters are bound to select only one category even when they have doubts. In contrast, fuzzy kappa allows computation of inter-rater agreement when the raters are allowed to express their understanding of the data by selecting multiple categories; in the above-mentioned example, the raters would be able to select both “coniferous forest” and “deciduous forest” categories. That is, while weighted kappa deals with one-to-one mapping, fuzzy kappa allows dealing with one-to-many mapping.

In conclusion, the paper proposes an indicator, fuzzy kappa, to measure inter-coder agreement in one-to-many content analysis protocol. It argues that one-to-many protocol has its uses in certain situations and for certain data types. The paper demonstrates that visual images, in particular, lend themselves well to “holistic” coding and, thus, represent one area of applicability of fuzzy kappa. The proposed approach to calculate inter-coder agreement builds on previous developments to construct fuzzy indices in natural sciences, primarily geography and biomedicine. Fuzzy kappa is an extension of Cohen’s kappa that uses fuzzy mathematics to access inter-coder agreement when classification into multiple categories is allowed. Both crisp and fuzzy measures return identical values when used with one-to-one classification or when the weight of the subsequent coder choices for the fuzzy indicator is set to zero. The approach proposed in this paper can be utilized with other crisp reliability indices, producing fuzzy pi (π), fuzzy alpha (α), etc. The fuzzy indicator can also be extended to the case of multiple coders, following the procedure suggested by Zuehlke [26] for fuzzy kappa in biological sciences. The fuzzy indices in general and fuzzy kappa proposed in this paper in particular need to be used with caution: the utility and applicability of fuzzy indicators require further investigation and their statistical properties need to be tested in various data domains.

## 6. Software

The software to calculate fuzzy kappa is freely available online at https://sourceforge.net/projects/fuzzy-kappa. To execute the program, run the batch file; by default, it will process Peru image data described in Example 2. The download includes all required libraries and presents a Windows-executable installation package of approximately 7 Mb in size. Instructions of how the coding data have to be presented are also provided.

## Supporting Information

S1 Zip Archive Russia photography classification data. (ZIP)

S2 Zip Archive Peru photography classification data. (ZIP)
